# 

**Published:** 1887-01-29

**Authors:** 


**The Hospital.**

**An Institution, Family, and Parochial Journal of Hospitals, Asylums, and all Agencies for the Care of the Sick, Criticism and News.**

SATURDAY, JANUARY 29TH, 1887.

### The Literary Prize.

A PRIZE of **Two Guineas** will be given every month for the best story or incident based upon hospital, or asylum, or student life, or any incident connected with the professions of medicine or nursing.

### Special Notice.

We shall publish a Special Supplement of eight pages in next week's Hospital, entitled a Revolution in Hospital Management, being the Report of the Special Committee on the Newcastle Infirmary.

### THE PRIZE COMPETITOR.

AT the request of many of our readers, we have determined to institute, in lieu of THE HOSPITAL "Charity Box," a

**Quarterly Prize Competition.**

We shall set each week two competitions, the nature of which we propose to vary from week to week, and we shall award at the end of each quarter, a

**Prize of Three Guineas**

to the Competitor who shall have sent in the largest number of correct or best answers to the PRIZE COMPETITOR. No one will be permitted to win the Prize twice in any one year, but we shall award annually an additional

**Prize of Five Guineas**

to the competitor who shall have sent in the largest number of correct or best answers during the twelve months. We hope this feature of THE HOSPITAL will prove one of interest and amusement to our numerous readers, and that it will be very generally participated in.

**No. 3. Play on Words.**

Translate and explain the play on the words in the following. (N.B.—We confess we cannot stand sponsors for the purity of the "Latin"). Theodoro Hooko uno die cum amico ambulante, "Ecce!" exclamavit amicus, "hominem quem cognosco et qui se tetotallerum appellat, potu inebriatum," "Nimirum," inquit Hookus, "nam scis quod ipse cum te tipse sit."

**No. 4. "Doctor."**

Give, in prose or verse, the best definition of "Doctor" which occurs to you. Answers may be droll, humorous, severe, or anything you like. Credit will be given for originality of thought, point, and terseness of expression. No reply should exceed one hundred words.

Answers must be addressed to THE PRIZE COMPETITOR, THE HOSPITAL, Norfolk House, Norfolk Street, Strand, W.C., not later than Saturday, 5th prox. The full name and address must in all cases be given, but a *nom de plume* may be added for publication. Only one side of the paper should be written on. The "Prize Competitor" Coupon (see advertisement pages) must be enclosed with replies.

### THE CHILDREN'S CORNER.

### OUR PASTIMES.

**Children's Quarterly Prize Competition.**

| | |
|---|---|
| Began | 1st Jan., 1887 |
| Ends | 26th Mar., 1887 |

PRIZES.

| | |
|---|---|
| 1st | Thirty Shillings |
| 2nd | Twenty " |
| 3rd | Ten " |
| 4th | Five " |

will be awarded to the four competitors who shall have sent in, during the thirteen weeks, the largest number of correct answers to our pastimes.

**Special Letter Prize.**

Each competitor is invited to forward a little letter, with the answers, addressed to the Puzzle Editor. The letters sent by competitors may be upon any subject they choose, such as their studies, their little pets, their little brothers, sisters or companions, little stories or recollections about the hospitals, or anything else they please. The letters should *not be too long*, and attention should be paid to the handwriting and the grammar. Only one side of the paper should be written on. The Puzzle Editor has decided to award a SPECIAL LETTER PRIZE OF TEN SHILLINGS for the best letters contributed during each Quarterly Competition.

**Special Prize for Neatness.**

A friend has kindly promised to hand the Puzzle Editor a Quarterly Prize of FIVE SHILLINGS for the *neatest answers* sent in. We shall have much pleasure in awarding this little prize.

**Fifth Competition.**

No. 28. CONUNDRUM. What is the riddle of riddles?

No. 29. CHARADE.

My *first* is no disgrace to tell,
Without my *second* you cannot spell:
My *whole* will help you to a wife,
Good sir, to bless you all your life.

No. 30. BURIED PROVERB. B r d s f f t h r f l c k t g t h r.

No. 31. CONUNDRUM. What vessel that is used by a chemist, most resembles an answer?

No. 32. LATIN PUZZLE. Who can render into "English," Dic fores superas vela nam.

**Results of Third Competition.**

No. 16. REBUS. The *centre* of gra-v-ity, is of course, the letter "v."

No. 17. GEOGRAPHICAL PUZZLE. First, Maid; second, Stone; whole, Maidstone.

No 18. BEHEADALS. The answers here are Four, *our*; when, *hen*; cat, *at*.

No. 20. REBUS. The most sensible letters in THE HOSPITAL, are, of course, the "y's" ("wise.") A.C.K. and others suggest the "y z's" ("the wiseheads.")

No. 21. HISTORICAL ENIGMA. By inserting a semi-colon after "talked," the sentence will make sense.

No. 22. HIDDEN WORDS. My last line revealed my secret, the answers giving a general chorus of "out."

*Six right*—Skye, Scrooge, Tim, Ellie, Joe, Mikado, Dot, A. C. K.; *Five right*—Florrie, Alsie, Prospect, Paul Methuen P. S.; *Four right*—Isobel, Little Mary, Filomel, Bismarck, Adriadne.

**Letters from Little Folk.**

My dear little friends have again sent me a charming little pile of correspondence, and I am only too sorry that my limited space prevents my printing it in full. No one has, thus far, written me about a hospital; I should so much like to hear from some on this subject.

Ellie writes me:

DEAR MR. EDITOR,—I have persuaded a companion at school to have THE HOSPITAL, and I think she will begin to answer the puzzles.—Yours faithfully, ELLIE.

44, Burgoyne Road, Brixton.

Scrooge has been very festive:—

DEAR MR. EDITOR,—My aunt gave a party last Friday, which I enjoyed immensely. The weather was very foggy, so unfortunately, some of the visitors did not come, but, nevertheless, we had a very jolly time. After tea, we played "Blind Man's Buff," and "Dumb Crambo," and then we danced the rest of the evening. When we were hot, my cousins and I played a "Toy Symphony," which we are going to play at a "Penny Concert" for poor people on Saturday. I will find some more puzzles to send next week.—Yours truly, SCROOGE.

7, Charles Street, Pendleton.

Here is a letter from the little daughter of an M.D.;—

DEAR EDITOR,—I do like your puzzles in THE HOSPITAL. They have been such fun since I began to do them on the first of January. I have holidays just now, so there is plenty of time to do them. They were very easy this week; no trouble at all. I think last week's ones were very difficult, especially the "Medical Medley." I thought nothing of THE HOSPITAL before I began your puzzles in January. We all look out so anxiously for it now; I hope you will never stop putting them in. I am afraid the last letter I wrote was too long. This one is not so long.—I remain, yours truly, SKYE.

4, Milton Terrace, New Road, Chatham.

**Letter Prize.**

One mark each: Skye, Scrooge, Tim, Isobel, Florrie, Ellie, Mikado, T. K., H. G. B., P. S., Little Mary, and Filomel.

**Notices to Correspondents.**

P.S.—Many thanks for the conundrums.

FLORRIE.—I am glad to hear you like the CHILDREN'S CORNER so much. Your little letters are capital. The promised puzzles will be most acceptable.

ISOBEL.—The two puzzles you sent me are very nice ones, and I thank you kindly for them.

H. G. B.—Thank you for your interesting little letter. I am glad your sister was pleased with her prize; I hope to see you answer the puzzles also. Please write on only one side of the paper.

T. K.—Your riddles are very amusing. How is it I have received no answers from you for the competition just commenced.

MIKADO.—Your wish that "THE HOSPITAL children" might be able to do something for those poor little children who are, as you say, "so cruelly treated," is our wish too. In London there is a Society for the Prevention of Cruelty to Children, and in some other towns there are similar agencies. Our best way to assist in the protection of little children who are cruelly used, is to help such societies as these—especially the London one.

Answers, having the actual name and address (to which a pseudonym may be added for publication), must have "The Children's" Coupon attached (see advertisement pages), and be addressed to the "PUZZLE EDITOR," THE HOSPITAL, Norfolk House, Norfolk Street, Strand, W.C., not later than Thursday next.

### NOTICE TO CORRESPONDENTS.

All correspondence must be written on one side of the paper only.

We cannot undertake to return MSS. not used.

**Communications,** whether intended for insertion or for private information, must be authenticated by the names and addresses of the writers, not necessarily for publication.

**Local papers** containing reports or news paragraphs **should be marked.**

It is especially requested that early intelligence of **local events of interest,** or which it may be thought desirable to bring under notice, may be sent direct to the Editor.

Communications respecting editorial matters should be addressed to the Editor, Norfolk House, Norfolk Street, Strand, London, W.C.

### THE IN- AND OUT-PATIENTS' HOSPITAL DIARY.

This Diary is so arranged as to make some portion of it appear every week, and the whole in each monthly part. It has been very difficult to compile, and corrections will at all times be gratefully received. It has been suggested that similar information about the larger Provincial and Scotch Hospitals would be useful to many readers. We should be glad to hear if this is felt to be the case.

### DISEASES AND IRREGULARITIES OF THE TEETH.—*continued.*

| Hospitals and Terms of Admission. | Physicians, Surgeons, and Medical Officers, with Days and Hours of Attendance. |
|---|---|
| New Hospital for Women, 222, Marylebone rd., W. | *Surgeon*, Mr. H. Apperley |
| North-West London Hosp., 18, Kentish Town Rd., N.W. | *Surgeon*, Mr. W. A. Maggs, F. 9 |
| Paddington Green Children's Hospital, Paddington | *Surgeon*, Mr. C. Vincent Cotterell, W. 9 |
| Royal Free Hospital, Gray's Inn Road, W.C. | *Surgeon*, Mr. H. Harris, M. Th. |
| Royal Hospital for Children and Women, Waterloo Bridge Road, S.E. | *Surgeon*, Mr. Whitehouse, F. 1.30 |
| St. Bartholomew's Hospital, Smithfield, E.C. | *Surgeons*, Messrs. Ewbank, Tu.; Paterson, F.; Ackery, F.; Mackrett, Tu. Hour, 9 |
| St. George's Hospital, Hyde Park Corner, S.W. | *Surgeon*, Mr. Winterbottom, Tu. S. 9 |
| St. Mary's Hospital, Cambridge Place, Paddington | *Susgeon*, Mr. H. Hayward, W. S. 9.30 |
| St. Thomas's Hospital, Westminster Bridge Road, S.E. | *Surgeon*, Mr. Truman, Tu. F. 10 |
| University College Hospital, Gower Street, W.C. | *Surgeon*, Mr. S. J. Hutchinson, W. 9.30 |
| Victoria Hospital for Children, Chelsea, S.W. | *Surgeon*, Mr. F. Fox, S. 9.0 |
| West London Hospital, Hammersmith Road, W. | *Surgeon*, Mr. H. L. Albert, Tu. F. 9.30 |
| Westminster Hospital, Broad Sanctuary, S.W. | *Surgeons*, Messrs. J. Walker and M. A. Smale, W. S. 9.15 |

### THROAT DISEASES.

| Hospitals and Terms of Admission. | Physicians, Surgeons, and Medical Officers, with Days and Hours of Attendance. |
|---|---|
| Central London Throat and Ear Hospital, Gray's Inn Road, W.C. | *Surgeons. See* "Ear Cases." M. W. Th. S. 2.30; Tu. F. 5 |
| Great Northern Central Hospital, Caledonian Rd, N. | *Surgeon*, Mr. Spencer Watson, W. 2 |
| Guy's Hospital, St. Thomas Street, S.E. | *Surgeon*, Mr. Symonds, Th. 1 |
| Hospital for Diseases of the Throat and Chest, 32, Golden Sq. Hon. Sec., Mr. G. C. Witherby. Poor free; others by small payment | *Physicians*, Drs. Wolfenden, Tu. F. 2.30; Macdonald, Tu. F. 6.30; Bond, W. S. 2.30. *Surgeon*, Mr. T. Mark Hovell, M. Th. 2.30 |
| King's College Hospital, Portugal Street, W.C. | *Surgeon*, Mr. Cartwright, Tu. F. 10 |
| Middlesex Hospital, Berners Street, W. | *Surgeon*, Mr. Hensman, Tu. 9 |
| North West London Hospital, 18, Kentish Tn. Rd., N.W | *Surgeon*, Mr. Collier, M. 1.30 |
| St. Bartholomew's Hospital, Smithfield, E.C. | *Surgeon*, Mr. Butlin, F. 2.30 |
| St. George's Hospital, Hyde Park Corner, S.W. | *Surgeon*, Dr. Whipham, Th. 1.30 |
| St. Mary's Hospital, Cambridge Place, Paddington | *Surgeon*, Mr. A. T. Norton, M. Th. 9.30 |
| St. Thomas's Hospital Westminster Bridge Road, S.E. | *Physician*, Dr. Semon, Tu. F. 1.30 |
| University College Hospital, Gower Street, W.C. | *Physician*, Dr. Poore, Th. 1.30 |
| Westminster Hospital, Broad Sanctuary, S.W. | W. 9. *See* General Hospitals. |

### DISEASES OF WOMEN, (OBSTETRIC CASES, ETC.)

| Hospitals and Terms of Admission. | Physicians, Surgeons, and Medical Officers, with Days and Hours of Attendance. |
|---|---|
| Charing Cross Hospital, West Strand, W.C. | *Physicians*, Drs. J. W. Black, Amand Routh, Tu. F. 1.30 |
| Chelsea Hospital for Women, Fulham Road, S.W. Sec., Mr. J. H. Easterbrook. Payment from 10*s.* 6*d.* a week. Poor by Governor's letter. Out-patients, if without subscriber's letter, pay 6*d.* each attendance | *In-Physicians*, Drs. J. H. Aveling, M. Th.; A. W. Edis, Tu. F.; F. Barnes, W. S. Hour, 2.30. *Out-Physicians* Drs. Dickinson and Mackeron, M.; W. Travers and G. Harper, Tu.; F. Jones and J. I. Parsons, W.; and H. T. Rutherford, F. Hour, 2 |

### DISEASES OF WOMEN, (OBSTETRIC CASES, ETC.)—*Continued.*

| Hospitals and Terms of Admission. | Physicians, Surgeons, and Medical Officers, with Days and Hours of Attendance. |
|---|---|
| East London Hospital for Children and Dispensary for Women, Shadwell, E. A Subscriber's letter is required, except in urgent cases | *Out-Physicians*, M. Tu. W. Th. F. 1 and 3; Tu. F. 9. *See* "Children's Diseases." |
| German Hospital, Dalston Lane, Dalston, E. | *Physician*, Dr. Rasch, M. Th. 9 |
| Great Northern Central Hospital, Caledonian Rd., N. | *Physician*, Dr. F. Barnes, W. 2 |
| Grosvenor Hospital for Women and Children, 3 and 4, Vincent Square, S.W. | *Out-Physicians and Surgeons*, Tu. W. Th. S. 2. *See* "Children's Diseases." |
| Guy's Hospital, St. Thomas Street, S.E. | *Physicians*, Drs. Galabin, Horrocks, M. Tu. F. 1.30 |
| Hospital of St. John and St. Elizabeth, 47, Gt. Ormond Street, W.C. For females suffering from long standing disease. | *Physicians*, Drs. Constable and Tegart (no out-patients) |
| Hospital for Women, 29 and 30, Soho Sq., W. Sec., Mr. David Cannon. In-patients admitted free by Governor's letter; also by payment. Out-patients free | *In-Physicians*, Drs. Carter, W. S. 2; R. T. Smith, Tu. F. 2; E. Holland, Tu. F. 9.30. *In-Surgeon*, Mr. H. A. Reeves, M. 4, Th. 2; *Out-Physicians*, Drs. R. T. Smith, F. 10; E. Holland, W. 10; Mansell-Moullin, M. Th. 11; Bedford Fenwick, Tu. 10; J. Oliver, S. 10. *Out-Surgeon*, Mr. S. Osborn, Th. 2 |
| King's College Hospital, Portugal Street, W.C. | *Physician*, Dr. Playfair, Tu. Th. S. 2 |
| London Hospital, Whitechapel Road, E. | *In-Physician*, Dr. Herman, Tu. F. 1.30. *Out-Physician*, Dr. Lewers, W. S. 1.30 |
| Metropolitan Free Hospital, Gray's Inn Road, W.C. | *Physician*, Dr. A. Venn, W. 2 |
| Middlesex Hospital, Berners Street, W. | *In-Physician*, Dr. Edis, Tu. F. 1.30. *Out-Physician*, Dr. Duncan, W. S. 1.30 |
| New Hospital for Women, 222, Marylebone Road, W. Hon. Sec., Miss C. Vincent. By subscriber's letter. Out-patients pay 6*d.* entrance fee, and 2*d.* each visit. | *In-Physicians*, Drs. Anderson, Atkins, Marshall, de la Cherois. *Out-Physicians*, Drs. Marshall, De la Cherois, Hitchcock. Daily, 1 to 3, and S 9 to 10. (All the physicians are women) |
| North-West London Hospital, 18, Kentish Tn. Rd., N.W | W. 1.30. *See* General Hospitals |
| Royal Free Hospital, Gray's Inn Road, W.C. | *In- and Out-Physician*, Dr. Hayes, Tu. S. 9 |
| Royal Hospital for Children and Women, Waterloo Bridge Road, S.E. Sec., Mr. R. G. Kestin. | *Out-Physicians*, Daily at 2. *Out-Surgeon*, W. 2. *See* "Children's Diseases." |
| St. Bartholomew's Hospital, Smithfield, E.C. | *In-Physician*, Dr. M. Duncan, Tu. Th. 2. *Out-Physician*, Dr. Godson, W. S. 9 |
| St. George's Hospital, Hyde Park Corner, S.W. | *Physician*, Dr. Champneys, Th. 1.30 |
| St. Mary's Hospital, Cambridge Place, Paddington, W. | *Physicians*, Drs. Meadows, Tu. F. 9.30; H. Jones, M. Th. 1.30 |
| St. Thomas's Hospital, Westminster Bridge Road, S.E. | *Physician*, Dr. Jervis, Tu. F. 2 |
| Samaritan Free Hospital for Women and Children, Lower Seymour Street, W. Branch, 1, Dorset Street, Manchester Square, W. Sec., Mr. Geo. Scudamore. Governor's letter or card is required if the disease of the applicant is *not* peculiar to her sex. Out-patients department (free) is at Edward's Mews, Duke Street, W. | *In-Physicians*, Drs. P. Boulton, M. Prickett, Amand Routh. Daily, 2. *In-Surgeons*, Messrs. G. G. Bantock, J. H. Thornton, W. A. Meredith. Daily, 9.30. *Out-Physicians*, Drs. M. Prickett, and Amand Routh, W. S. 1.30. *Out-Surgeons*, Messrs. W. A. Meredith, and J. D. Malcolm, M. Th.; A. H. G. Doran, and W. S. A. Griffith, Tu. F. Hour 1.30 |
| University College Hospital, Gower Street, W.C. | *Physicians*, Drs. G. Hewitt, M. Th. 1.30; J. Williams, Tu. F. 2 |
| West London Hospital, Hammersmith Road, W. | *Physicians*, Drs. A. Venn, Moullin, W. S. 2 |
| Westminster Hospital, Broad Sanctuary, S W. | *Physicians*, Drs. Potter, Tu. F. 2; Grigg, Tu. F. 9 |

